# Conjugated linoleic acid influences the metabolism of tocopherol in lactating rats but has little effect on tissue tocopherol concentrations in pups

**DOI:** 10.1186/s12944-016-0272-x

**Published:** 2016-05-31

**Authors:** Johanna O. Zeitz, Erika Most, Klaus Eder

**Affiliations:** University of Giessen, Institute of Animal Nutrition and Nutritional Physiology, Heinrich-Buff-Ring 26-32 (IFZ), D-35392 Giessen, Germany

**Keywords:** Conjugated linoleic acid, Lactating rat, Vitamin metabolism, Tocopherol

## Abstract

**Background:**

Conjugated linoleic acid (CLA) is known to affect the lipid metabolism in growing and lactating animals. However, potential effects on the metabolism of fat-soluble vitamins in lactating animals and co-occurring effects on their offspring are unknown. We aimed to investigate the effects of dietary CLA on concentrations of tocopherol in various tissues of lactating rats and their offspring and expression of genes involved in tocopherol metabolism.

**Methods:**

Twenty-eight Wistar Han rats were allocated to 2 groups and fed either a control diet (control group) or a diet containing 0.9 % of cis-9, trans-11 and trans-10, cis-12 (1:1) CLA (CLA group) during pregnancy and lactation. Feed intake of dams and body weight of dams and their pups were recorded weekly. Tocopherol concentrations in various body tissues were determined at day 14 of lactation in dams and 1, 7 and 14 days after birth in pups. Expression of selected genes involved in metabolism of tocopherol was determined in dams and pups. The data were statistically analysed by analysis of variance.

**Results:**

Feed intake and body weight development of nursing rats and their pups was similar in both groups. In livers of CLA-fed dams, tocopherol concentrations decreased by 24 % but expression of TTPA and CYP3A1, involved in tocopherol transport and metabolism, were not influenced. In the dams’ adipose tissue, gene expression of receptors involved in tissue tocopherol uptake, LDLR and SCARB1, but not of LPL, increased by 30 to 50 % and tocopherol concentrations increased by 47 % in CLA-fed compared to control dams. Expression of LPL, LDLR and SCARB1 in mammary gland was not influenced by CLA-feeding. Tocopherol concentrations in the pup’s livers and lungs were similar in both groups, but at 14 days of age, adipose tissue tocopherol concentrations, and LDLR and SCARB1 expression, were higher in the CLA-exposed pups.

**Conclusions:**

We show that dietary CLA affects tissue concentrations of tocopherol in lactating rats and tocopherol metabolism in rats and pups, but hardly influences tissue tocopherol concentrations in their offspring. This indicates that supplementation of CLA in pregnant and lactating animals is uncritical considering the tocopherol status of new-borns.

## Background

Dietary CLA supplements, which usually consist of a mixture of the cis-9, trans-11 CLA and the trans-10, cis-12 CLA isomer, are known to influence the lipid metabolism during lactation and growth in different species like rats [1, 2], pigs [3] and ruminants [4, 5]. The CLA influence the lipid metabolism by down regulating gene expression of proteins involved in lipid synthesis and fatty acid (FA) transport in many tissue like muscle, adipose tissue, liver [1, 3] and mammary gland [4, 6]. Consequently, the FA composition of tissues and milk is altered by CLA [1, 4, 5]. Similarly, CLA might influence the metabolism of tocopherols, which is linked to the lipid metabolism concerning absorption and intermediary metabolism [7]. For example, tocopherols are emulsified by bile acids in the intestine and transported in chylomicrons and lipoproteins together with other lipids [7]. Likewise, the lipoprotein lipase (LPL) is not only crucial for uptake of triglycerides (TG) and FA from chylomicrons and very low density lipoproteins (VLDL) into extra-hepatic tissues [8], but it is also important for uptake of tocopherols [7]. Some first studies in growing animals indicate that feeding CLA in fact influences tissue tocopherol concentrations. For example, in the liver of mice and rats, levels of α-tocopherol increased when CLA was supplemented to the diet [9, 10]. The molecular mechanism underlying these observations are largely unknown but may be related to increased levels of the α-tocopherol transfer protein (TTPA) which is responsible for retaining α-tocopherol in the body [9]. Increased concentrations of α-tocopherol in plasma and adipose tissue due to CLA feeding have been found as well [9, 10]. Tocopherol has not only important functions as an antioxidant but also e.g., in cell signalling and inflammation [7]. When changes in tocopherol status due to CLA feeding occur, this may affect not only the animal fed CLA, but, in case of pregnant and lactating animals, also growth and development of their offspring. However, studies investigating the tocopherol metabolism in response to CLA feeding in lactating animals and potential effects on their offspring are missing so far. We hypothesized that dietary CLA influences the tocopherol status of lactating rats and their offspring. Therefore, we determined tocopherol concentrations in various tissues of dams and their pups. Furthermore, we aimed to reveal the molecular mechanisms underlying the observed changes in tissue tocopherol status by investigating gene expression of selected enzymes and cell membrane receptors involved in the metabolism of tocopherol.

## Methods

### Animal experiment

Twenty-eight female Wistar Han rats were obtained from Harlan Laboratories (AN Venray, The Netherlands) at their 2^nd^ day of pregnancy. The rats were randomly assigned to 2 groups of 14 rats each, with an initial body weight of 220 ± 16.2 g (mean ± SD; Control group) and 218 ± 18.5 g (CLA group) (*P* = 0.75). The pregnant rats were housed in groups of 2 animals each in Macrolon cages (type 4) enriched with cellulose papers and a cardboard tube in a room controlled for temperature (22 ± 2 °C), relative humidity (55 ± 10 %), ventilation (20/h), and light (12-h-light/-dark cycle, <200 Lux). Water was available *ad libitum* from nipple drinkers during the whole experiment. Bedding was exchanged every week. Experimental diets were fed during pregnancy and lactation. The rats were fed semipurified diets meeting their nutrient requirements according to the recommendations of the National Research Council for the AIN-93G diet [11] (Table 1). Titanium dioxide (TiO_2_) was added at 0.5 % of the diet to determine the apparent total tract digestibility of tocopherol. The CLA supplement Lutalin® (BASF, Ludwigshafen, Germany), which contained 29 % each of the cis-9, trans-11 and the trans-10, cis-12 CLA isomer (data not shown), replaced part of the soybean oil and was supplemented in a dosage of 15 g/kg diet in the CLA group (Table 1). In the control group, linoleic acid rich sunflower oil (15 g/kg diet) replaced part of the soybean oil to approximate dietary FA other than linoleic acid and CLA in both experimental diets. The CLA supplement contained 12.8 g of total CLA/100 g of dietary FAs which is equivalent to 0.9 g CLA/100 g of diet. Diets were pelleted to 10 mm with a standard pelleting device (Kahl Laborpressanlage Typ 14–175, Reinbek, Germany). Feed was offered *ad libitum* and intake was recorded weekly. After parturition, rats were individually housed together with their pups. Litters were adjusted to 8 pups per dam without differentiation of gender after a maximum of 3 days after parturition. Two dams from the control group were removed from the experiment because 1 dam devoured the whole litter directly after parturition and 1 dam had to be killed due to problems with delivery. All experimental procedures were in strict accordance with the Appendix A of European Convention for the Protection of Vertebrate Animals used for Experimental and other Scientific Purposes (ETS NO. 123). In Accordance with Article 4 par. 3 of the German Animal Welfare Law all animals were humanely killed for scientific purposes which was approved by the Animal Welfare Officer of the Justus-Liebig-University, JLU No. 480_M.

**Table 1:** Ingredients and concentrations of individual tocopherols in the experimental diets

	Control diet	CLA diet
Ingredient, g/kg diet		
Corn starch	532	532
Casein	200	200
Sucrose	100	100
Cellulose	50	50
Soybean oil	55	55
Sunflower oil	15	-
CLA supplement (Lutalin®)	-	15
Mineral mix^a^	35	35
Vitamin mix^a^	10	10
L-Cysteine	3	3
Dietary tocopherol concentration (μmol/kg diet)^b^		
α-tocopherol	140 ± 3.28	141 ± 2.02
γ-tocopherol	60.4 ± 1.30	59.8 ± 1.43
δ-tocopherol	6.57 ± 0.37	6.58 ± 0.22
Tocopherol equivalents^c^	147 ± 3.28	147 ± 2.01

^a^The mineral and vitamin mix were composed as recommended for the AIN 93G diet by Reeves et al. [11]

^b^means ± standard deviations of 5 analyses of feed samples collected in weeks 1 to 5. The β- and δ-tocopherols, which are quantitatively negligible as source of active vitamin E in soybean and sunflower oil [32], could not be separated by HPLC and are summarized as δ-tocopherol

^c^sum of active α, δ- and γ-tocopherol considering conversion factors of 0.10 for γ-tocopherol and 0.03 for δ-tocopherol as compared to α-tocopherol (100 % active) [17]

### Sample collection

Feed samples were taken weekly and stored at −20 °C for later tocopherol and FA analysis. Feed intake and weights of rats and their litter were recorded weekly. Within 24 h after birth, 2 randomly chosen pups/litter were decapitated and liver, lung, and the milk curd from the stomach were removed. After 7 days, 1 pup/litter was anesthetized with isoflurane, decapitated, and liver, lung, subcutaneous fat and the milk curd from the stomach were removed. At day 13 of lactation, the dam’s feces, which had been excreted during the preceding 24 h, was collected and stored at −20 °C. At day 14, i.e., at peak lactation and before weaning [12, 13], all dams and the remaining pups were decapitated under anaesthesia with carbon dioxide. From the pups, liver, lung, subcutaneous fat and the milk curd from the stomach were removed. Blood from the dams was collected into heparinised polyethylene tubes (Sarstedt) and plasma was separated by centrifugation (1100 × *g*, 10 min) at 4 °C and stored at −20 °C. From the dams, liver, mammary gland (right), gastrocnemius muscle (left) and kidney fat (left) were excised. All tissue samples were immediately snap-frozen in liquid nitrogen and stored at −80 °C pending further analysis.

### Determination of tissue lipid content and of fatty acid composition

For milk curd fat determination, about 0.2 g of lyophilized milk curd was dispersed with 3 mL of 0.9 % NaCl and the fat was extracted with 4 mL hexane: isopropanol (HiP, 3:2, v/v) [14] overnight. After centrifugation at 1000 × *g* for 5 min at 15 °C, the upper fat-containing phase was quantitatively transferred to a dried and weighed test glass and the fat extraction procedure was repeated once. The test glass was dried to constant weight at 105 °C and reweighed and the weighed fat was related to the initial milk curd weight. Feed and liver FA composition was determined according to Schlegel et al. [5]. In brief, 0.25 g of milled feed or dietary oil or 50 mg of homogenized liver tissue was extracted with 4 or 0.5 mL of HiP (3:2, v/v), respectively, excluding light overnight on a shaker. After centrifugation (10 min at 15 °C and 1000 × g), 250 (feed), 50 (oil) or 100 μL (liver) of the upper fat-containing phase was evaporated to dryness under N_2_ at 37 °C and methylated with 50 (feed, liver) or 150 μL (oil) of trimethylsulfonium hydroxide [15]. The fatty acid methyl esters were separated by gas chromatography using a PE Clarus 580 system (Perkin Elmer, US) equipped with an automatic split injector, a polar capillary column (50 m, 0.25 mm i.d., 0.2 μm film thickness; Macherey and Nagel, Düren, Germany), and a flame ionization detector. Helium was used as carrier gas (flow rate 1.2 mL/min). The individual fatty acid methyl esters were identified by comparing their retention times with those of purified standards. The Δ-9 and Δ-6 desaturase indices were calculated to estimate desaturase activities according to Masek et al. [16].

### Determination of tocopherols

Concentrations of tocopherols in feed, feces, plasma, tissue and milk curd were determined by high-performance liquid chromatography (HPLC) (L-7100, LaChrom, Merck-Hitachi, Darmstadt, Germany). Samples of 0.1 g of feed, feces, homogenized tissue or milk curd or of 0.2 mL of plasma, were mixed with 2 mL of a 10 g/L pyrogallol solution (in ethanol, absolute) and 300 μL of a saturated sodium hydroxide solution. After flushing with N_2_, this mixture was heated for 30 min at 70 °C in closed glass tubes. The tocopherols were then extracted by addition of 2 mL of n-hexane and 2 mL of bidest. water. After centrifugation (5 min at 10 °C and 1000 × g), an aliquot of the hexane phase was evaporated to dryness under N_2_ and re-dissolved in methanol containing 0.05 % of butylated hydroxytoluene. The tocopherols were separated isocratically by HPLC using methanol as mobile phase, a C18 column (≥3 μm particle size, 4 mm length, 2 mm internal diameter, Phenomenex, Germany) (pre-column) and a C-18-reversed phase column (Luna C18 (2), 150*4.6 mm; Phenomenex, Aschafffenburg, Germany) and detected by fluorescence (Fluorescence Detector L-7480, LaChrom, Merck-Hitachi). Excitation and emission wavelengths for tocopherols were 295 and 325 nm, respectively. Tocopherol equivalents were calculated using the stereoisomer of α-tocopherol, RRR α-tocopherol, as a reference (RRR α-tocopherol = 1), and γ-tocopherol was multiplied by 0.10 and δ-tocopherol by 0.03 to consider the lower vitamin activity of those 2 tocopherol isomers [17].

### Determination of tocopherol digestibility

The apparent total tract digestibility of tocopherol was determined using TiO_2_ as an indigestible marker. The TiO_2_ concentrations in feed and feces were measured photometrically according to Brandt and Allam [18] and the digestibility was calculated as: apparent total tract digestibility = 100 – {(% TiO_2_ in diet/% TiO_2_ in feces) × (% tocopherol in feces/% tocopherol in diet) × 100}.

### RNA isolation and Quantitative real-time RT-PCR

Total RNA was isolated from 20 mg of liver tissue (dams, pups) using Trizol reagent (Invitrogen) according to the manufacturer’s protocol and from 80 mg of kidney fat (dams) or subcutaneous fat (pups), or from 40 mg of mammary gland tissue (dams) using the RNeasy Lipid Tissue Mini Kit (Qiagen, Hilden, Germany). The RNA concentration and purity were estimated from the optical density at 260 and 280 nm, respectively, using an Infinite 200 M microplate reader and a NanoQuant Plate (both from Tecan, Männedorf, Switzerland) and RNA was stored at −80 °C. The cDNA was synthesized from 1.2 μg of total RNA using 100 pmol dT18 primer (Eurofins MWG Operon, Ebersberg, Germany), 1.25 μL 10 mmol/L dNTP mix (GeneCraft, Lüdinghausen, Germany), 5 μL buffer (Fermentas, St. Leon-Rot, Deutschland), and 60 units M-MuLV Reverse Transcriptase (MBI Fermentas, St. Leon-Rot, Germany) at 42 °C for 60 min, and a final inactivating step at 70 °C for 10 min in Biometra ThermalCycler (Whatman BiometraW, Göttingen, Germany). The cDNA was diluted 1:2 with DNase/RNase free water and stored at −20 °C. The qPCR was carried out on a Rotorgene 2000 system (Corbett Research, Mortlake, Australia) using 2 μL cDNA combined with 9 μL of a mixture composed of 5 μL KAPA SYBR FAST qPCR Universal Mastermix (Peqlab, Erlangen, Germany), 0.2 μL each of 10 μM forward and reverse primers and 3.6 μL DNase/RNase free water in 0.1 mL tubes (Ltf Labortechnik, Wasserburg, Germany). Gene-specific primer pairs were from [19] or were designed using PRIMER3 and BLAST and obtained from Eurofins MWG Operon (Ebersberg, Germany) (Table 2). The amplification of a single product of the expected size was approved using 2 % agarose gel electrophoresis stained with GelRed™ nucleic acid gel stain (Biotium Inc., Hayward, CA). The Ct values of target and reference genes were obtained using Rotorgene Software 5.0 (Corbett Research). All Ct values were transformed into relative quantification data using the 2^-ΔCt^ equation [20] but the efficiencies of the reference and target genes were determined and used for the calculations instead of using an efficiency of 2. The highest relative quantities for each gene were set to 1. These expression values of target genes were normalized using the GeNorm normalization factor [21]. Using the Microsoft Excel-based application GeNorm, the GeNorm normalization factor was calculated as the geometric mean of expression data (relative quantities) of the three most stable out of four to six tested potential reference genes (ATP5B, CANX, MDH1, RPL13, TOP1, YWHAZ for liver and fat; ACTB instead of ATP5B for mammary gland). The normalized expression values data set was corrected for outliers. Means and SD were calculated from normalized expression data for samples of the same experimental group. The mean of the control group was set to 1 and the means and SD of the CLA group was scaled proportionally.

**Table 2:** Characteristics of primers used for qPCR

Gene	GeneBank accession no.	Sense primer (5′-3′)	Antisense primer (5′-3′)	Product size, bp	Primer efficiency	Annealing Temp., °C
Reference genes						
ACTB	NM_031144.2	GACCTCTATGCCAACACAGT	CACCAATCCACACAGAGTAC	154	1.92	60
ATP5B	NM_134364.1	GCACCGTCAGAACTATTGCT	GAATTCAGGAGCCTCAGCAT	203	1.90	60
CANX	NM_172008.2	CCAGATGCAGATCTGAAGAC	CTGGGTCCTCAATTTCACGT	175	2.31	60
MDH1	NM_033235.1	CAGACAAAGAAGAGGTTGCC	CGTCAGGCAGTTTGTATTGG	206	1.97	60
RPL13	NM_031101.1	CTTAAATTGGCCACGCAGCT	CTTCTCAACGTCTTGCTCTG	198	1.94	60
TOP1	NM_022615.1	GAAGAACGCTATCCAGAAGG	GCTTTGGGACTCAGCTTCAT	137	2.00	60
YWHAZ	NM_013011.3	GACGGAAGGTGCTGAGAAA	GCAGCAACCTCAGCCAAGT	198	2.09	60
Target genes						
TTPA	NM_013048.2	GGAGGTGGAAACTCAACGGAA	AGCAGCAATCTTCTTGGCTACA	109	1.93	60
CYP3A1	NM_013105.2	TGGTAATAGACTTGAGAGAG	GGGCAGATATACATAAGGA	196	1.90	56
LDLR	NM_175762.2	ACAGTGTCCTCCCAAGTCCAA	GCAAATGTGGATCTCGTCCTC	222	1.87	60
SCARB1	NM_031541.1	GGTGCCCATCATTTACCAAC	CCCTACAGCTTGGCTTCTTG	162	1.94	60
LPL	NM_012598.2	GAGATTTCTCTGTATGGCACA	CTGCAGATGAGAAACTTTCTC	276	1.99	60

Primers were designed using Primer3 from gene sequences obtained from GeneBank and tested *in silico* using BeaconDesigner and mfold. Primer specificity was determined using a BLAST search

### Statistics

Data were analysed by one-way analysis of variance using the software R version 3.1.3 (https://cran.r-project.org). For analysis of data with measurements at only one time point, treatment was considered as fixed and animal as random effect and the function lme, package nlme, was used. Time-series data were monitored over time; the model included treatment, time and their interaction as fixed effects and animal as random effect using lmer; pairwise comparisons were done using the Hochberg test and the function lsmeans.

## Results

### Tocopherol and fatty acid composition of the diet

The concentration of tocopherols were similar in both diets (Table 1). The FA composition of the experimental diets is shown in Table 3. As intended, the FA composition of the 2 experimental diets was similar and differed only in their contents of CLA and linoleic acid (Table 3).

**Table 3:** FA composition of the control and CLA-supplemented diet (g/100 g of total FAs)^a^

	Control diet	CLA diet
C 16:0	10.04 ± 0.02	10.04 ± 0.06
C 18:0	3.22 ± 0.04	3.38 ± 0.07
C 18:1 n9	22.51 ± 0.21	22.91 ± 0.09
C 18:1 n7	1.37 ± 0.01	1.38 ± 0.01
C 18:2 n6	57.51 ± 0.17	45.04 ± 0.32
C 18:3 n3	4.43 ± 0.11	4.46 ± 0.09
C18:2 cis-9, trans-11 CLA	<0.1	6.04 ± 0.21
C18:2 trans-10, cis-12 CLA	<0.1	5.82 ± 0.21
C 20:0	0.25 ± 0.01	0.27 ± 0.01
C 20:1	0.19 ± 0.01	0.21 ± 0.01
C 20:5 n3	0.42 ± 0.02	0.41 ± 0.02

^a^mean ± standard deviation of 5 analyses for diets (samples of week 1–5)

### Effects of CLA on performance, liver lipids, tocopherol digestibility, and tissue tocopherol concentration of dams

Feed intake was similar in control and CLA-fed rats, but increased after delivery with the onset of lactation in both groups (Table 4). Consequently, the dietary intake of tocopherols increased with the onset of lactation in week 4 but was similar between groups (Table 4). Body weight development of dams before and after delivery was not influenced by the supplementation of CLA to the diet (Table 4). After 5 experimental weeks, at peak lactation, dam liver weights (% of body weight) were similar in both groups, however, liver TG concentrations were lower in CLA-fed dams compared to control dams (Table 5). The FA composition of total liver lipids differed between groups (Table 5). As expected, the concentrations of both CLA isomers and of stearic acid were higher in the CLA group as compared to the control group while the concentrations of mono- and polyunsaturated C18 FAs including linoleic acid were lower (Table 5). In addition, Δ-9 and Δ-6 desaturase indices, i.e., the estimated desaturase activities, were lower in the CLA-fed groups compared to the control group (Table 5). The apparent digestibility of tocopherol was considerably lower in the CLA-fed dams than in the Control dams (Table 6). Still, concentrations of tocopherols in plasma and muscle of dams were not influenced by CLA feeding (Table 6). However, the tocopherol concentration in the liver decreased by 24 % in the CLA-fed dams whereas that in the adipose tissue increased by 47 % (Table 6). Both fat and tocopherol concentrations in the milk curd were similar in both groups (Table 7). Tocopherol concentrations in milk curd were highest one day after delivery and decreased during the first 2 weeks of lactation (Table 7).

**Table 4:** Feed and tocopherol intake and body weight of dams fed either a control or a CLA-supplemented diet^d^

	Week					SEM	*P* value		
	1	2	3	4	5		Treatment	Time	tr.*time
Feed intake, g/d									
Control	21.7^a^	20.8^a^	21.9^a^	25.2^b^	43.2^c^	0.57	0.43	<0.001	0.25
CLA	21.1^a^	20.4^a^	20.8^a^	26.5^b^	43.8^c^				
Tocopherol intake, μmol/d^e^									
Control	3.18^a^	3.05^a^	3.21^a^	3.69^b^	6.33^c^	0.08	0.99	<0.001	0.40
CLA	3.09^a^	2.99^a^	3.05^a^	3.88^b^	6.41^c^				
Body weight, g									
Control	258^a^	292^b^	286^b^	294^b^	289^b^	4.9	0.95	<0.001	0.75
CLA	256^a^	293^b^	283^b^	289^b^	285^b^				

^a-c^Means with superscripts without a common letter differ at *P* < 0.05. ^d^Values are means, *n* = 12–14. Means with superscripts without a common letter differ at *P* < 0.05. Weeks 1–3: pregnancy, weeks 4–5: lactation; weight for week 3 is after delivery; tr., treatment. Data were analysed by two-way ANOVA with treatment, time and their interaction as fixed effects and animal as random effect

^e^sum of active α, δ- and γ-tocopherol considering conversion factors of 0.10 for γ-tocopherol and 0.03 for δ-tocopherol as compared to α-tocopherol (100 % active) [17]

**Table 5:** Liver weights, liver TG content and FA composition of total liver lipids of dams fed either a control or a CLA-supplemented diet (g/100 g of total FA)^a^

	Control	CLA	SEM	*P* value
Liver, g/100 g BW	5.05	5.12	0.16	0.70
Liver TG, μmol/g liver	111	84	12	0.028
C 14:0	1.17	1.24	0.086	0.45
C 16:0	29.7	30.1	0.84	0.69
C 16:1 n9	4.80	3.85	0.40	0.026
C 18:0	9.25	12.7	0.77	<0.001
C 18:1 n9	31.5	28.7	1.05	0.012
C 18:2 n6	15.1	12.7	0.87	0.010
C 18:3 n6	1.22	0.81	0.073	<0.001
C 18:3 n3	0.32	0.29	0.027	0.33
C18:2 cis-9, trans-11 CLA	<0.1	0.76	0.061	<0.001
C18:2 trans-10, cis-12 CLA	<0.1	0.33	0.033	<0.001
C 20:4 n6	5.67	7.10	0.61	0.028
C 22:6	1.16	1.52	0.18	0.053
Δ-9D 16 (16:1n9/16:0)^b^	0.16	0.13	0.011	0.0043
Δ-9D 18 (18:1n9/18:0)^b^	3.47	2.39	0.256	<0.001
Δ-6D n6 (18:3n6/18:2n6)^b^	0.08	0.07	0.004	<0.001

^a^Values are means at day 14 of lactation, *n* = 12–14. Data were analysed by one-way ANOVA with treatment as fixed effect and animal as random effect

^b^to estimate desaturase activities, desaturase indices were calculated as product-to precursor ratios of individual fatty acids. Δ-9D 16, Δ-9 desaturase index for C16:1n9, Δ-9D 18, Δ-9 desaturase index for C18:1n9, Δ-6D n6, Δ-6 desaturase index for the n6 line

**Table 6:** Apparent tocopherol digestibility and tocopherol concentration in plasma and tissues of dams fed either a control or a CLA-supplemented diet^a^

	Control	CLA	SEM	*P* value
Tocopherol digestibility, %	77.9	68.7	2.09	<0.001
Plasma and tissue tocopherol concentrations				
Plasma, μmol/l	22.1	24.5	1.44	0.12
Liver, nmol/g	491	374	27.5	<0.001
Adipose tissue, nmol/g	136	199	14.2	<0.001
Muscle, nmol/g	45.0	45.3	3.29	0.91

^a^Values are means at day 14 of lactation, *n* = 12–14. Data were analysed by one-way ANOVA with treatment as fixed effect and animal as random effect

**Table 7:** Litter development, milk curd fat content and tocopherol concentration in milk curd and tissues of pups nursed by dams fed either a control or a CLA-supplemented diet^d^

Day after birth	1		7		14			*P* value		
Treatment group	Control	CLA	Control	CLA	Control	CLA	SEM	Treatment	Time	tr.*time
Performance of pups										
Litter weight, g	56.4^a^	57.7^a^	143^b^	149^b^	264^c^	272^c^	3.35	0.36	<0.001	0.48
Pup weight, g	7.1^a^	7.2^a^	17.9^b^	18.6^b^	38.2^c^	39.2^c^	0.46	0.39	<0.001	0.60
Liver weights and liver triglyceride concentrations										
Liver, g/100 g BW	4.62^a^	4.80^a^	NA	NA	3.10^b^	3.32^b^	0.13	0.30	<0.001	0.92
Liver TG, μmol/g liver	32.6	48.5	NA	NA	40.9	36.7	3.28	0.21	0.69	0.032
Fat content										
Milk curd, μmol/g	299	312	327	292	329	301	6.40	0.059	0.51	0.015
Tocopherol concentration										
Milk curd, nmol/g	500^a^	429^a^	57.5^b^	55.6^b^	65.5^b^	59.8^b^	11.4	0.084	<0.001	0.077
Liver, nmol/g	381^a^	320^a^	121^b^	102^b^	102^b^	91^b^	19.2	0.21	<0.001	0.67
Lung, nmol/g	35.8^a^	39.3^a^	72.1^b^	67.9^b^	65.6^b^	71.0^b^	2.39	0.62	<0.001	0.24
Adipose tissue, nmol/g	NA	NA	52.2^a^	50.9^a^	49.0^a^	71.9^b^	2.46	0.001	0.002	<0.001

^a-c^Means with superscripts without a common letter differ at P < 0.05. ^d^Values are means after standardization to 8 pups per litter, *n* = 12–14. Means with superscripts without a common letter differ at *P* < 0.05. tr., treatment. NA, not analysed. Data were analysed by two-way ANOVA with treatment, time and their interaction as fixed effects and animal as random effect

### Effects of CLA on performance and tissue tocopherol concentration of pups

At delivery, litter weights were similar in the control (67.8 ± 14.0 g) and the CLA group (69.0 ± 16.3 g) (*P* = 0.85). Litter size did not differ between the control (11.2 ± 2.33 pups per litter) and CLA group (11.0 ± 3.06) as well (*P* = 0.88). All 26 rats used for data analysis delivered within 3 days and litter size was standardized to 8 pups per dam on average 1.1 days after birth (maximum 2 days after birth). Litter development after litter size standardization was similar in both groups (Table 7). Liver weights were similar between groups both in the newborn pups and in 14 day-old pups (Table 7). Likewise, weights of the lung were similar in pups nursed by CLA-fed dams (2.28 ± 0.42 g/100 g BW) and in pups nursed by Control dams (2.45 ± 0.62 g/100 g BW) (*P* = 0.42). The tocopherol concentration in livers and lungs of pups did not differ between the two groups, but were higher in new-born pups as compared to 7 and 14 day-old pups (Table 7). In the adipose tissue, concentrations of tocopherols were higher in the CLA group as compared to the control group in the 14 day-old pups, but did not differ in the 7-day old pups (Table 7).

### Effects of CLA on expression of genes involved in metabolism of lipids and tocopherols in liver, adipose tissue and mammary gland of dams and pups

**Fig. 1:**
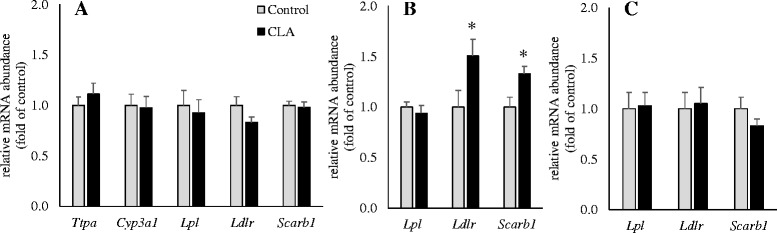
Relative mRNA abundance of genes involved in lipid and tocopherol metabolism in liver (**a**), adipose tissue (**b**) and mammary gland (**c**) of rats fed either a control or a CLA-supplemented diet. Bars represent mean ± SEM of 12 to 14 rats per group and are expressed as fold of relative mRNA abundance of the control group. *, different from the control group at *P* < 0.05. CYP3A1, cytochrome P450, family 3, subfamily A, polypeptide 1; LDLR, low density lipoprotein receptor; LPL, lipoprotein lipase; SCARB1, scavenger receptor class B, member 1; TTPA: α-tocopherol transfer protein

**Fig. 2:**
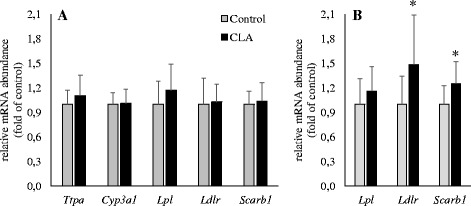
Relative mRNA abundance of genes involved in lipid and tocopherol metabolism in liver (**a**) and adipose tissue (**b**) of pups nursed by dams fed either a control or a CLA-supplemented diet. Bars represent mean ± SEM of 12 pups per group and are expressed as fold of relative mRNA abundance of the control group. *, different from the control group at *P* < 0.05. CYP3A1, cytochrome P450, family 3, subfamily A, polypeptide 1; LDLR, low density lipoprotein receptor; LPL, lipoprotein lipase; SCARB1, scavenger receptor class B, member 1; TTPA: α-tocopherol transfer protein

In the liver of dams at day 14 of lactation, the gene expression of TTPA was similar in both groups, and the CLA supplement did also not influence the expression of CYP3A1, a cytochrome P450 enzyme which is important for tocopherol degradation in rat liver [22, 23] (Fig. 1a). In the adipose tissue, the gene expression of LDLR and SCARB1, which are involved in uptake of lipoproteins and lipid-soluble vitamins, were enhanced by 50 and 30 %, respectively, in the CLA group as compared to the control group, but the expression of LPL was similar (Fig. 1b). In liver and mammary gland, the gene expression of LDLR, SCARB1 and LPL was similar between groups (Fig. 1a and 1c). Similarly, in the pup’s liver, gene expression of TTPA, CYP3A1, LDLR, SCARB1 and LPL were not influenced by CLA feeding (Fig. 2a). However, in the adipose tissue, gene expression of LDLR and SCARB1 were enhanced in the pups nursed by CLA-fed dams compared to those nursed by control dams (Fig. 2b).

## Discussion

In our study, feed intake and growth of the pregnant and lactating rats was not influenced by dietary supplementation of 0.9 g of total CLA/100 g of diet. This has been observed by others as well when CLAs were supplemented to rat diets in dosages between 0.25 and 1.35 g CLA/100 g of diet [2, 24, 25]. Like in the present study, pup growth has been found unchanged when rat diets were supplemented with 0.25 to 0.5 g CLA/100 g of diet [2, 24]. On the other hand, when higher dietary CLA concentrations of 1.35 to 1.47 g CLA/100 g of the dams’ diet have been used, pup growth as well as the dams’ milk fat contents was reduced [1, 25]. The observation of our study that the fat contents of the milk curd did not differ between groups fits well to the finding that pup growth was not influenced by CLA feeding of the dams and may be related to the use of lower CLA dosages as compared to the studies of Ringseis et al. [1] and Hayashi et al. [25]. As expected, the CLA concentrations in liver lipids were higher in the CLA group as compared to the control group, which confirms that CLA was incorporated into body lipids. Also, the concentrations of oleic and linoleic acid and of gamma-linoleic acid in liver lipids and the Δ-9 and Δ-6 desaturase indices decreased which can be explained by the t10,c12-CLA-induced inhibition of the stearoyl-CoA desaturase (SCD; Δ-9 desaturase) and the fatty acid desaturase 2 (FADS2; Δ-6 desaturase), respectively [26, 27].

In the dams, our hypothesis that CLA affect body concentrations of tocopherols was confirmed, because the concentrations of tocopherols were decreased in liver and increased in the adipose tissue. This partly confirms findings of Chao et al. [9] and Chen et al. [10] who reported increased concentrations of tocopherols in, amongst others, adipose tissue of growing mice. In general, increased tocopherol concentrations in the adipose tissue could be explained either by an accumulation of tocopherols due to reductions in fat mass or by enhanced deposition of tocopherols in the adipose tissue or by both processes. We didn’t measure adipose tissue mass in the present study and thus cannot exclude a reduction of adipose tissue mass due to CLA feeding as shown by Salgado et al. [28]. However, our data do suggest a reallocation of tocopherols from the liver, where tocopherol concentrations decreased, to the adipose tissue where tocopherol concentrations increased. The finding that liver tocopherol concentrations decreased are in contrast to other studies, which found increased liver tocopherol concentrations after CLA supplementation [9, 10]. Likewise, our data contrast with that of Chen et al. [10] where tocopherol digestibility was not influenced by CLA-feeding in mice, whereas we observed that tocopherol digestibility in CLA-fed dams was reduced compared to control dams. A reduced tocopherol absorption may help to explain reduced liver tocopherol concentrations due to CLA-feeding in the present study. Also, in contrast to others who have shown that gene expression of the TTPA in the liver of growing mice fed similar dosages of CLA (0.74–0.80 %) in the diet was enhanced [9, 10], the TTPA mRNA concentrations in the liver of the lactating dams in our study were not altered. The TTPA triggers association of α-tocopherol with lipoproteins and the secretion from the liver into the bloodstream [7]. It seems also likely that CLA supplementation did not influence tocopherol degradation in the present study, because gene expression of CYP3A1 was similar in both groups and the CYP-dependent ω-hydroxylation of tocopherols is the rate-limiting step in tocopherol degradation [23]. Liver and plasma concentrations of α-carboxyethyl hydroxychroman, the degradation product of tocopherol, excreted via urine and bile, have been unchanged by feeding dietary CLA in the study of Chen et al. [10] as well. However, our data indicate that plasma lipoproteins and thus also tocopherols were preferably taken up by the adipose tissue as the gene expression of LDLR and SCARB1, which are involved in uptake of lipids and tocopherols from lipoproteins [7], where enhanced in the adipose tissue, but not increased in liver and mammary gland. In all tissues investigated, gene expression of the LPL, which is involved in TG uptake from VLDL and chylomicrons [29], was not influenced by CLA. This was unexpected because in the mammary gland of both rat [1] and ruminants [4, 5], the LPL has been shown to be down-regulated upon CLA-feeding. Interestingly, the uptake of α-tocopherol is dependent upon LPL in the mammary gland of lactating rats, but not in adipose tissue [30]. This fits to our observation that tocopherol concentrations were enhanced in the adipose tissue although LPL gene expression was not. The latter is also in line with a study of Andreoli et al. [31], which has shown that the activity of the LPL in white adipose tissue was not influenced when 1.0 or 2.9 % of CLA were included into a rat diet.

The pups’ tocopherol intake was probably similar because the tocopherol concentration in the milk curd was similar in both groups. Also, our sampling was done at peak lactation, 14 days after birth, or before which is before onset of solid feed intake by pups occurs (~18 days after birth) [13], which was also confirmed by visual examination of the pups’ stomach contents in our study. Accordingly, the effects of CLA-feeding of the dams on the pups’ body tocopherol status was minor and limited to the adipose tissue at the last sampling time point. Only at this time point, at 14 days of age, pups nursing CLA-fed dams had higher adipose tissue tocopherol concentrations than those from the control group, and the increased gene expression of LDLR and SCARB1 indicate that tocopherol uptake into the pup’s adipose tissue was increased in pups nursed by CLA-fed dams compared to control dams. Still, we cannot exclude the possibility that the higher tocopherol concentration in the pups’ adipose tissue was partly due to a simple concentration effect: the CLA which can be transferred to the pups’ body via milk [1] may influence the pups’ lipid metabolism as well and reduce adipose tissue mass, which may in turn lead to enhanced tocopherol concentrations in the remaining adipose tissue mass. Together, this indicates that dietary CLA supplementation of pregnant and lactating rats in dosages similar to those in the present study do not influence milk tocopherol concentrations and hardly influence tocopherol status in pups.

## Conclusions

In conclusion, we demonstrate that dietary CLA influence tissue tocopherol status in lactating rats. We also present evidence that dietary CLA influence tocopherol metabolism on gene expression level. Based on our data, we conclude that CLA increases liver export of α-tocopherol and uptake of tocopherols from lipoproteins into the adipose tissue but not into mammary gland. We also show that CLA has only minimal effects on tissue tocopherol status of pups nursed by CLA-fed dams although it seems that the CLA transferred to pups via milk affect the pup’s tocopherol metabolism similar than it does in dams. These data indicate that supplementation of CLA in pregnant and lactating animals is uncritical in terms of tocopherol status of new-borns.

## Abbreviations

ACTB, actin, beta; ANOVA, analysis of variance; ATP5B, ATP synthase, H+ transporting, mitochondrial F1 complex, beta polypeptide; CANX, calnexin; CLA, conjugated linoleic acid; CYP3A1, cytochrome P450, family 3, subfamily A, polypeptide 1; FA, fatty acid; HiP, hexane: isopropanol; HPLC, high-performance liquid chromatography; LDLR, low density lipoprotein receptor; LPL, lipoprotein lipase; MDH1, malate dehydrogenase 1, NAD (soluble); RPL13, ribosomal protein L13; SCARB1, scavenger receptor class B, member 1; SCD, stearoyl-CoA desaturase; SD, standard deviation; TG, triglyceride; TOP1, topoisomerase (DNA) I; TTPA, α-tocopherol transfer protein; VLDL, very low density lipoprotein; YWHAZ, tyrosine 3-monooxygenase/tryptophan 5-monooxygenase activation protein, zeta.
